# Chitosan–Fucoidan Gel Formation for Food Packaging Film Development Incorporating Blackcurrant Anthocyanins for Monitoring Tuna Freshness

**DOI:** 10.3390/gels12060465

**Published:** 2026-05-27

**Authors:** Haofeng Wang, Nongawendé S. Gloria Saguin, Hao Lan, Jingrong Gao, Yadong Zhao, Shanggui Deng

**Affiliations:** 1 School of Food and Pharmacy, Zhejiang Ocean University, Zhoushan 316022, China; harvey_wang0420@163.com (H.W.); gloriasaguin21@gmail.com (N.S.G.S.); dengshanggui@163.com (S.D.); 2 School of Food Science and Technology, Dalian Polytechnic University, Dalian 116034, China; 3 Faculty of Food Science, Zhejiang Pharmaceutical University, Ningbo 315000, China; lanhao.888@163.com

**Keywords:** chitosan, fucoidan, anthocyanin, tuna, packaging

## Abstract

This study investigated the development, fabrication, and characterization of a novel biodegradable food packaging film based on chitosan (CH) and fucoidan (FU), incorporating blackcurrant-derived anthocyanins (BCAs). The system was designed to enable real-time monitoring of tuna (*Thunnus* spp.) freshness, while addressing environmental concerns through the replacement of synthetic materials with a bioactive, multifunctional alternative that provides both mechanical protection and dynamic spoilage indication. Films were prepared using a casting method with varying BCA concentrations (0.2%, 0.4%, and 0.6%) and systematically evaluated in terms of their structural, physicochemical, and biological properties. The results indicated that the CH/FU/BCA film containing 0.4% BCA exhibited optimal performance, characterized by enhanced tensile strength, reduced water solubility and moisture content, and improved thermal stability and barrier properties. The incorporation of BCA enabled distinct color changes in response to spoilage-related conditions, supporting its function as a pH-responsive indicator. In addition, the films demonstrated significant antimicrobial activity against *Escherichia coli* and *Staphylococcus aureus*, affirming their suitability as active packaging materials. Zeta potential analysis further indicated improved colloidal stability upon BCA incorporation. Overall, the synergistic interactions among CH, FU, and BCA resulted in a multifunctional film with combined protective and freshness-indicating capabilities. These findings highlight the potential of the developed biofilm for application in intelligent seafood packaging systems.

## 1. Introduction

Food packaging plays a critical role in protecting food from chemical, physical, and biological hazards throughout the supply chain. It is essential for mitigating various factors that affect food quality, including odors, mechanical shocks, dust, temperature, light, and humidity [1]. Plastics remain the predominant packaging material due to their excellent mechanical properties, barrier functions, and permeability characteristics. However, environmental worries have spurred research on renewable-source biodegradable packaging films [2,3]. Such films can be effectively fabricated using natural polymers, including carbohydrates, starch, cellulose, hemicellulose, and pectin.

Chitosan, obtained from chitin in crustacean shells, has attracted considerable attention as a biodegradable packaging material owing to its antimicrobial activity, film-forming ability, and cost-effectiveness [4]. Its intrinsic antimicrobial properties enhance water resistance and flexibility, making it suitable for food packaging applications. Moreover, chitosan’s biodegradability makes it a sustainable plastic alternative because of its biodegradability, biocompatibility, and functional versatility [5]. Structurally, chitosan is a linear polysaccharide composed of β-(1→4)-linked D-glucosamine and N-acetyl-D-glucosamine units, conferring excellent mechanical strength, flexibility, and barrier properties against oxygen and microorganisms [6]. Consequently, chitosan-based films have been explored extensively for food packaging and biomedical applications. For instance, Chitosan films incorporated with honeysuckle flower extract were investigated by Wang et al. for active food packaging applications [7], while Yilmaz et al. developed chitosan films infused with Ficus carica Linn leaf extract and characterized their properties [8]. Despite these advantages, pure chitosan films often exhibit limitations such as insufficient mechanical strength and poor water barrier properties, which can restrict their practical utility [9]. To overcome these shortcomings, chitosan is frequently combined with other biopolymers, such as gelatin, to produce composite films with improved performance [10]. Recent studies have demonstrated that incorporating gelatin, essential oils, phenolic compounds, polysaccharides, and various nanoparticles can significantly enhance the mechanical, barrier, and antimicrobial properties of chitosan-based films [11].

Fucoidan is a sulfated polysaccharide predominantly composed of L-fucose and sulfate ester groups, naturally occurring in brown seaweed [12]. It is highly valued for its antioxidant, anticoagulant, antimicrobial, and antiviral activities, and is utilized in a variety of food products and biomaterials [7]. The polyanionic nature of fucoidan, conferred by its sulfate and carboxyl groups, allows it to interact with proteins and metal ions, enhancing its chelating potential [13]. When combined with chitosan, fucoidan forms a polyelectrolyte complex through electrostatic interactions between oppositely charged groups: chitosan is cationic under acidic conditions, whereas fucoidan is anionic due to its sulfate and carboxyl groups [14,15].

This interaction improves multiple physicochemical properties of the complex, including solubility, pH responsiveness, thermal stability, and mechanical strength, rendering it suitable for applications in films, hydrogels, and nanoparticles [16,17]. Furthermore, the chitosan–fucoidan combination preserves biodegradability while exhibiting synergistic bioactivities such as antimicrobial, antioxidant, and anticoagulant effects [18]. Its pH-responsive behavior and biodegradability make it particularly advantageous for applications in drug delivery, wound healing, tissue engineering, and food packaging, leveraging the complementary characteristics of both biopolymers [19]. Despite these advantages, chitosan–fucoidan films may still suffer from limited mechanical strength and require intricate preparation procedures, as well as stability and durability challenges under varying environmental conditions [20]. To address these limitations, researchers frequently incorporate bioactive compounds with antibacterial and antioxidant properties into the films, enhancing their functional performance [21].

Polyphenols are widely employed in the development of polymer films due to their abundance and cost-effectiveness [22]. The inclusion of polyphenols into chitosan–fucoidan films has been found to reinforce their mechanical properties and barrier properties, thereby improving their suitability for food packaging applications [23]. Among polyphenols, anthocyanins—water-soluble natural pigments—are extensively utilized in intelligent packaging owing to their rich sources, low cost, strong antioxidant and antimicrobial activities, pronounced pH responsiveness, and high biological safety [24,25]. Blackcurrant (Ribes nigrum) serves as a prominent source of anthocyanins and other functional phytochemicals, including flavonoids and ascorbic acid, which confer antioxidant and antimicrobial functionalities [26,27].

Incorporation of blackcurrant extracts into polymer films enhances food preservation by scavenging free radicals and inhibiting lipid oxidation, particularly in fatty foods susceptible to spoilage, thereby maintaining both quality and nutritional value [28]. Furthermore, the pH sensitivity of blackcurrant anthocyanins enables the development of colorimetric sensors within packaging materials. For example, Yar et al. [29] developed a composite film of carboxymethyl cellulose, xanthan gum, and citric acid that leveraged blackcurrant anthocyanins to monitor beef spoilage. The films exhibited marked color changes in response to pH shifts and ammonia exposure, with ΔE values reflecting transitions from red-pink to black-brown, green, green-yellow, and yellow, providing a visual indicator of food freshness [29,30].

From a sustainability perspective, anthocyanin-based films offer biodegradable alternatives to conventional plastics, aligning with the growing consumer demand for environmentally friendly packaging [31]. Moreover, these smart packaging systems demonstrate enhanced antimicrobial activity when combined with biopolymers such as chitosan and fucoidan [32]. Blackcurrant anthocyanins thus represent versatile and promising agents for innovative food packaging, simultaneously addressing concerns related to food quality, safety, and environmental impact, and offering significant potential for further development in the food industry.

Due to improving living standards, home meal replacement (HMR) products have seen a sharp rise in demand worldwide [33]. Tuna, a popular HMR option, is valued for its high protein content but remains susceptible to microbial contamination under ambient storage conditions [34,35]. Intelligent packaging has emerged as an effective strategy for enhancing the fish product safety and shelf stability [36]. Accordingly, this study aimed to formulate an intelligent packaging system utilizing chitosan and fucoidan in combination with anthocyanins extracted from blackcurrant, for the purpose of monitoring tuna freshness. The novelty of this work lies not only in evaluating how anthocyanin incorporation enhances the functional properties of chitosan films but also in investigating the electrostatic interactions between chitosan and fucoidan. Since limited studies have focused on the development of pH-responsive chitosan-fucoidan composite films incorporated with blackcurrant anthocyanin for seafood freshness monitoring, this study aimed to fabricate intelligent packaging films with enhanced pH sensitivity, barrier performance and antimicrobial activity. Unlike previous reports that mostly use single biopolymer matrices or anthocyanins from common sources, we introduce fucoidan as a reinforcing polyelectrolyte and use blackcurrant anthocyanins specifically for tuna freshness monitoring, which remains underexplored. For this purpose, structural, morphological, thermal, mechanical, hydrophobic, antibacterial and tuna preservation analyses were systematically conducted to evaluate the physicochemical and functional properties of the prepared films.

## 2. Results and Discussion

### 2.1. Characterization of Anthocyanin

**Figure 1:**
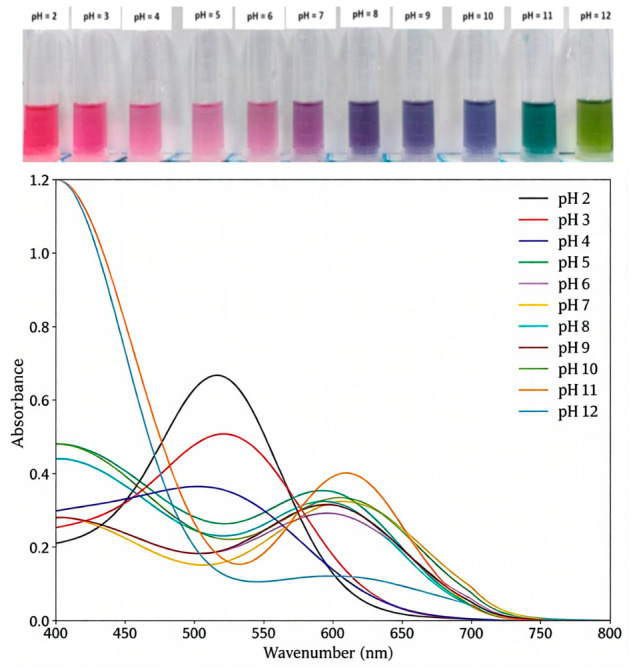
UV vis spectra of anthocyanins.

The blackcurrant anthocyanin (BCA) content was determined to be 250.8 ± 2.2 mg/g. The color variations observed in BCA solutions, along with their corresponding UV–visible absorption spectra, arise from the conformational shifts of anthocyanins under different pH environments [37]. At pH 2, the solution exhibited an orange-red color, which shifted to red at pH 3 due to the predominance of flavylium cations. As the pH increased from 4 to 6, the red coloration gradually diminished, corresponding to the conversion of flavylium cations into colorless carbinol forms through hydration reactions. When the pH ranged from 7 to 9, the solution exhibited a purple coloration, reflecting the formation of anionic quinoidal bases. Further increases in pH (10–12) resulted in a color transition from green to yellow, which was associated with the transformation of quinoidal bases into chalcone structures. The UV–visible absorption spectra of BCA solutions (Figure 1) further supported these observations. At pH values below 7, a characteristic absorption peak was observed at approximately 550 nm, corresponding to the red flavylium cation. As the pH increased from 2 to 6, the intensity of this peak progressively decreased, indicating the hydration of flavylium cations into colorless carbinol species and the consequent reduction in red color intensity. Similar behavior has been reported by Yan et al., who developed chitosan-based films incorporating anthocyanin-rich Robusta coffee peel extract [38]. At pH values above 7, the absorption maximum shifted to approximately 600 nm, which was caused by the development of purple/blue quinoidal base structures. These findings demonstrate the pH-responsive structural transitions of anthocyanins, which govern both the color variation and spectral characteristics of BCA [39]. Such transformations are reversible and are primarily driven by protonation, deprotonation, and hydration equilibria, consistent with the observed color and spectral changes.

### 2.2. FTIR and XRD Analysis

**Figure 2:**
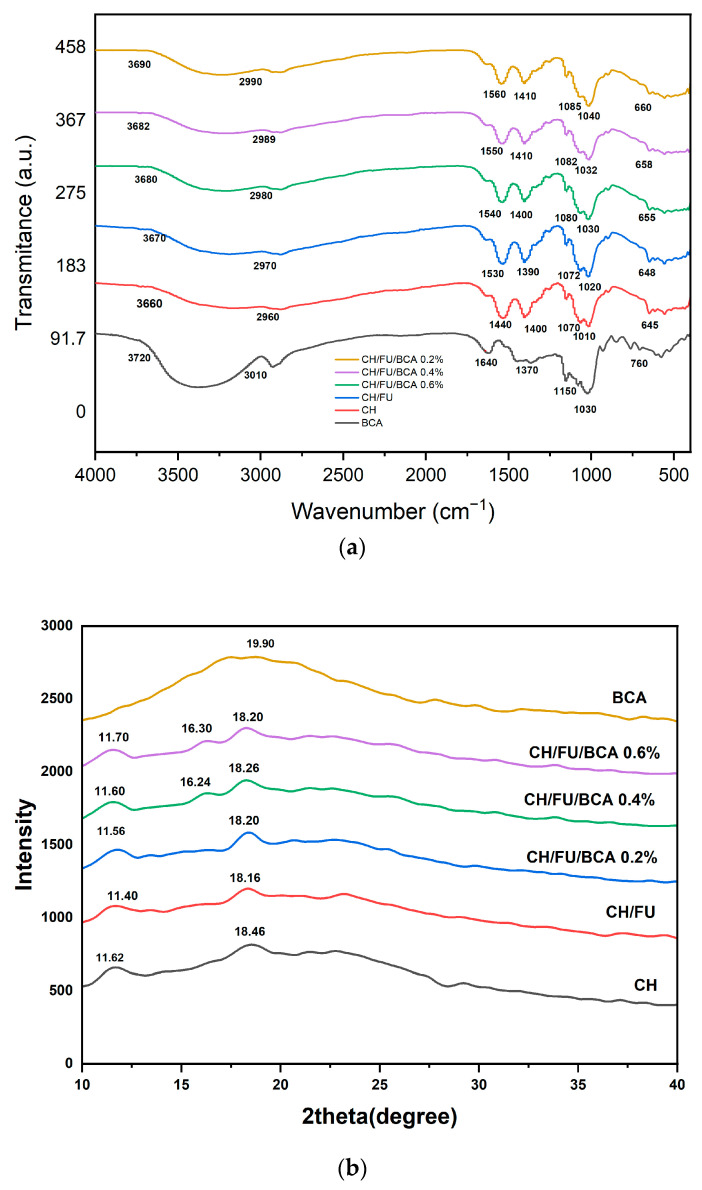
FTIR spectra (**a**) and X-ray diffraction (XRD) patterns (**b**) of chitosan (CH), CH/FU, and CH/FU/BCA films containing 0.2%, 0.4%, and 0.6% BCA.

FTIR spectroscopy was employed to evaluate the interactions among the film components, including chitosan, fucoidan, and blackcurrant anthocyanins (BCA), as shown in Figure 2a. For BCA, the broad absorption band at 3720 cm^−1^ was attributed to O–H stretching vibrations, while the peak at 3010 cm^−1^ corresponded to C–H stretching. The absorption band at 1640 cm^−1^ was associated with C=O stretching vibrations, whereas the peaks observed at 1150 cm^−1^ and 954 cm^−1^ were assigned to C–O stretching vibrations.

The X-ray diffraction (XRD) patterns of the films are presented in Figure 2b. BCA exhibited a broad amorphous diffraction peak centered at 19.90°, indicating its predominantly amorphous nature. In contrast, chitosan displayed semicrystalline characteristics, with distinct diffraction peaks observed at 11.62° and 18.46°.

FTIR analysis revealed the presence of intermolecular interactions, particularly hydrogen bonding. The characteristic absorption bands corresponding to C=O and C–O vibrations remained evident, indicating the structural integrity of blackcurrant anthocyanins within the composite system [40]. For chitosan (CH), the broad band at 3660 cm^−1^ was attributed to O–H and N–H stretching vibrations, while the peak at 2960 cm^−1^ corresponded to C–H stretching. The absorption band at 1440 cm^−1^ was associated with C–N stretching, and the peak at 1010 cm^−1^ was assigned to C–O stretching vibrations.

Upon the incorporation of fucoidan, the O–H/N–H stretching band shifted from 3660 cm^−1^ (CH) to 3670 cm^−1^ (CH/FU), verifying the formation of hydrogen bonding and electrostatic interactions between the two polysaccharides. With the further addition of BCA at concentrations ranging from 0.2% to 0.6%, this band progressively shifted from 3680 to 3690 cm^−1^, indicating enhanced intermolecular interactions derived from hydrogen bonding between the hydroxyl moieties of BCA and the amino/hydroxyl functionalities of chitosan. Comparable findings have been documented by Wang, F. et al. who observed comparable spectral shifts upon incorporating anthocyanins from coleus leaves into chitosan-based composite films [41].

XRD analysis demonstrated that chitosan exhibited a semicrystalline structure, whereas blackcurrant anthocyanins displayed an amorphous profile. The XRD patterns of the CH/FU/BCA composite films showed only minor variations in peak intensity and position compared with the CH/FU matrix, indicating that the incorporation of BCA did not markedly alter the crystalline structure of the films. However, BCA may have enhanced intermolecular interactions within the composite system, such as hydrogen bonding and possible non-covalent associations, which are likely to contribute to improved mechanical stability and barrier properties while maintaining film flexibility, thereby supporting their potential application in food packaging. Previous studies on polysaccharide–anthocyanin systems confirm these findings, which emphasize the importance of intermolecular interactions in determining functional performance. Overall, the results suggest that hydrogen bonding plays a dominant role in structuring CH/FU/BCA films without the necessity for covalent crosslinking, providing a foundation for the development of bioactive packaging materials.

### 2.3. Morphology

**Figure 3:**
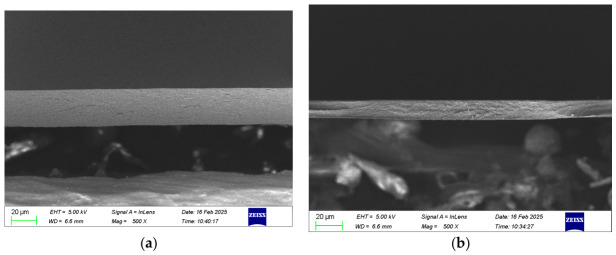

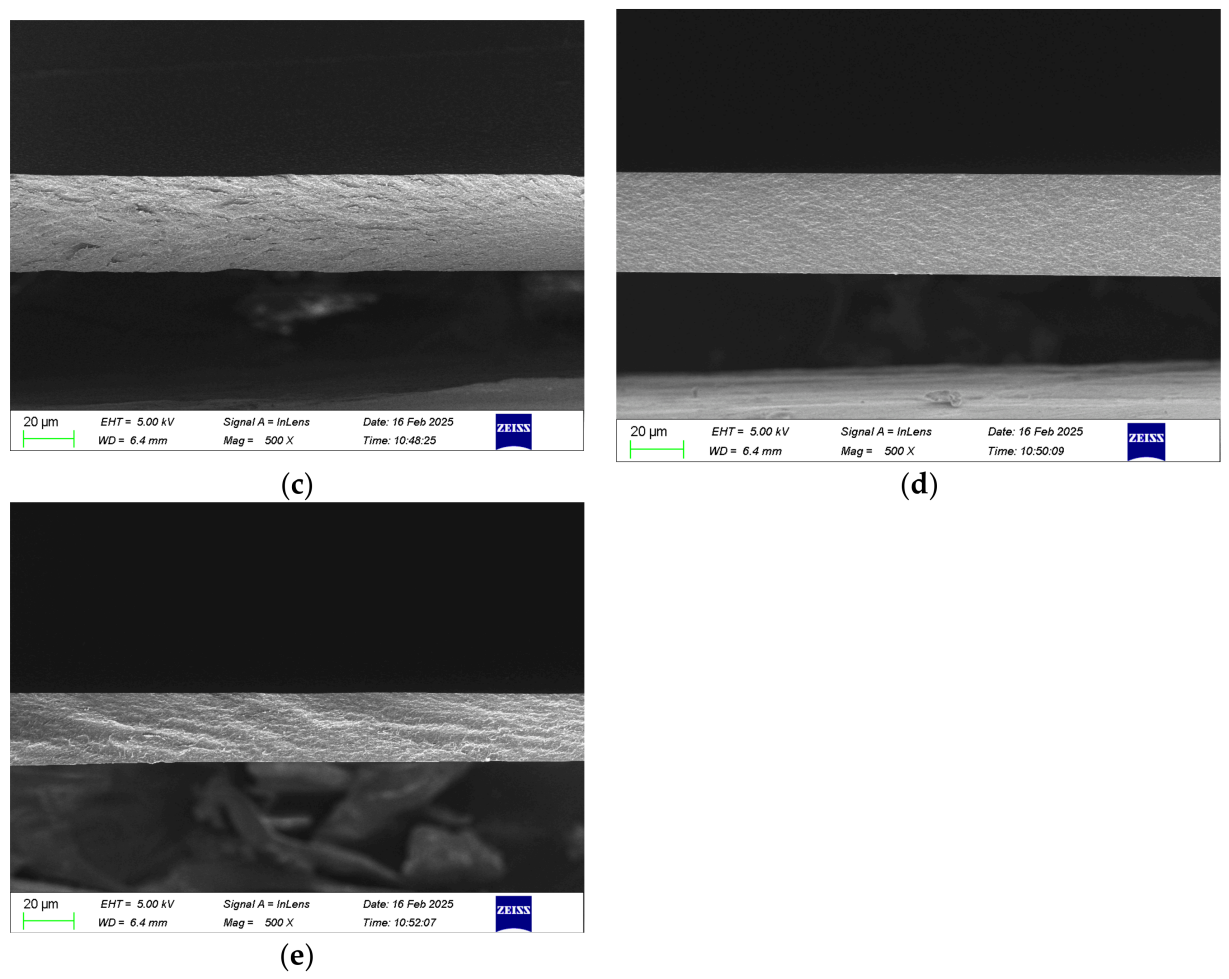
Cross-sectional morphology of chitosan (CH) (**a**), CH/FU (**b**), and CH/FU/BCA composite films containing 0.2% (**c**), 0.4% (**d**), and 0.6% (**e**) BCA.

Figure 3 presents the cross-sectional microstructural characteristics of the films. The pure chitosan (CH) film exhibited a smooth and homogeneous cross-section without visible wrinkles or defects. In contrast, the incorporation of BCA resulted in the development of a more porous structure within the composite films.

Chitosan (CH), chitosan–fucoidan (CH/FU), and BCA-incorporated composite films exhibited distinct morphological variations, reflecting the interactions among their components. The pure CH film displayed a smooth and compact surface, which can be attributed to the strong intermolecular hydrogen bonding within the chitosan matrix. Upon the incorporation of fucoidan, the CH/FU films exhibited a more porous and irregular structure, suggesting disruption of the ordered hydrogen-bonding network of chitosan by the amorphous fucoidan [42]. This structural loosening is likely to enhance permeability and may be advantageous for applications requiring controlled release. The addition of BCA further modified the film morphology in a concentration-dependent manner. At 0.2% BCA, only slight changes in surface structure were observed, with a marginal increase in uniformity compared to the CH/FU film, indicating the initial formation of intermolecular interactions that partially reduced porosity. Increasing the BCA content to 0.4% (Figure 3d) resulted in more pronounced structural changes, characterized by a smoother and more homogeneous morphology, suggesting enhanced intermolecular interactions and improved structural integrity. At the highest concentration (0.6% BCA, Figure 3e), the most significant morphological modification was observed, indicating a further strengthening of the film network.

At higher BCA concentrations, the films exhibited a more compact and smoother morphology, indicating enhanced intermolecular interactions and reduced porosity. While such structural densification is expected to improve mechanical properties, it may concurrently reduce permeability. The incorporation of BCA, particularly at 0.4% and 0.6%, likely enhanced the mechanical strength of the films through increased intermolecular interactions, thereby improving their durability and suitability for applications requiring structural integrity. SEM observations further demonstrated that BCA significantly influenced the surface morphology of chitosan–fucoidan films. Pure chitosan films exhibited a dense and uniform structure, whereas the addition of fucoidan increased porosity and surface irregularity [43]. With increasing BCA content, the film surfaces became progressively smoother and more homogeneous, which can be attributed to strengthened intermolecular interactions within the composite matrix. These structural modifications are expected to have a considerable impact on the mechanical, barrier, and functional properties of the films. The present findings are consistent with previous studies on anthocyanin–polysaccharide systems, which have established that bioactive compound concentration critically governs both film architecture and functional properties [44].

### 2.4. Thermal Characteristic Analysis

**Figure 4:**
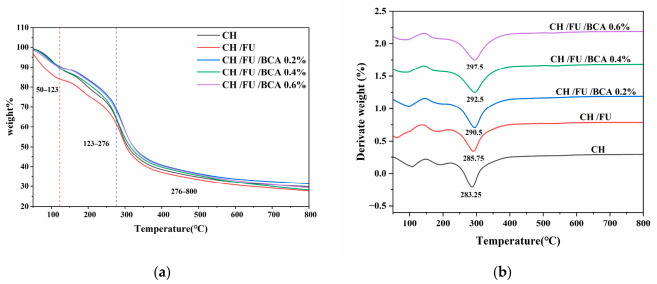
Thermogravimetric (TGA) curves (**a**) and derivative thermogravimetric (DTG) curves (**b**) of CH, CH/FU, and CH/FU/BCA film samples.

The effect of anthocyanin incorporation on the thermal stability of the films was evaluated using thermogravimetric analysis (TGA). The corresponding TGA and derivative thermogravimetric (DTG) curves are presented in Figure 4. As shown in Figure 4a, the thermal degradation of the chitosan-based composite films occurred in three distinct stages: the first stage (50–123 °C), the second stage (123–276 °C), and the final stage (276–800 °C). The DTG curves (Figure 4b) further indicated that the incorporation of fucoidan and BCA shifted the main degradation peaks toward higher temperatures and decreased the maximum degradation rate, suggesting an improvement in the thermal stability of the composite films.

Thermogravimetric analysis (TGA) indicated that chitosan (CH) and its composite films underwent thermal degradation in three distinct stages. The initial stage (50–123 °C) was primarily associated with the evaporation of physically adsorbed water and residual acetic acid. The second stage (123–276 °C) corresponded to the degradation of functional groups, including amino and hydroxymethyl moieties [45]. In the final stage (276–800 °C), the weight loss noted was ascribed to the breaking down of the chitosan/fucoidan backbone and the aromatic structures present within the chitosan–BCA composites. At 800 °C, the residual weight of the pure CH film was 27.45%, whereas the chitosan–fucoidan (CH/FU) composite exhibited a slightly higher residue of 28.22%, suggesting that the incorporation of fucoidan contributed to enhanced thermal resistance. Furthermore, the addition of BCA led to a progressive increase in residual mass, ranging from 29.65% for CH/FU/BCA (0.2%) to 31.40% for CH/FU/BCA (0.6%), indicating that BCA incorporation further bolstered the thermal resistance of the films. The differential thermogravimetric (DTG) curves supported these findings, showing a shift in the main degradation peaks toward higher temperatures along with a reduction in the maximum degradation rate upon the addition of fucoidan and BCA. These results collectively demonstrate that the composite films exhibited enhanced thermal stability compared to pure chitosan, likely due to strengthened intermolecular interactions and the formation of a more compact network structure.

### 2.5. Thickness, Moisture Content (MC), and Water Solubility (WS)

The moisture content (MC), thickness, and water solubility (WS) of CH, CH/FU, and CH/FU/BCA films are presented in Table 1. Different lowercase letters indicate significant differences among the means within the same column (*p* < 0.05). The thickness of the films ranged from 0.029 ± 0.001 mm to 0.038 ± 0.001 mm, suggesting that the incorporation of fucoidan and BCA did not significantly affect film thickness. Film thickness is primarily influenced by the composition and the relative concentrations of the components within the film-forming matrix.

The incorporation of anthocyanins contributed to the formation of a more rigid film structure, indicating effective entrapment of anthocyanins within the biopolymer matrix [46]. Similarly, Li et al. reported that the addition of anthocyanins, including those incorporated via nanoparticles, did not significantly affect film thickness [47]. In terms of moisture content, the pure chitosan film exhibited the highest value (16.7 ± 0.056%), which can be attributed to the abundance of hydrophilic functional groups [31]. The incorporation of fucoidan reduced the moisture content to 11.4 ± 0.080%. Furthermore, the addition of anthocyanins resulted in a progressive decrease in moisture content, reaching 10.4 ± 0.042%, 9.5 ± 0.040%, and 8.4 ± 0.037% at 0.2%, 0.4%, and 0.6%, respectively. This reduction is likely associated with decreased availability of free hydrophilic hydroxyl groups and the formation of a more compact film matrix, thereby limiting water retention.

A similar trend was observed for water solubility. The pure chitosan film exhibited the highest solubility (16.22 ± 0.020%), which decreased to 12.34 ± 0.002% upon incorporation of fucoidan. The addition of BCA further reduced water solubility, with values of 11.38 ± 0.002%, 11.29 ± 0.002%, and 7.02 ± 0.001% for 0.2%, 0.4%, and 0.6% BCA, respectively. This reduction suggests enhanced intermolecular interactions within the film matrix, contributing to improved structural integrity. Overall, the decreases in moisture content and water solubility indicate enhanced water resistance and suggest potential improvements in mechanical performance, supporting the suitability of these films for food packaging applications. These findings are consistent with previous studies reporting that the incorporation of fucoidan and anthocyanins effectively reduces moisture sensitivity and solubility, thereby enhancing film stability in humid environments [48,49].

### 2.6. Mechanical Properties

**Figure 5:**
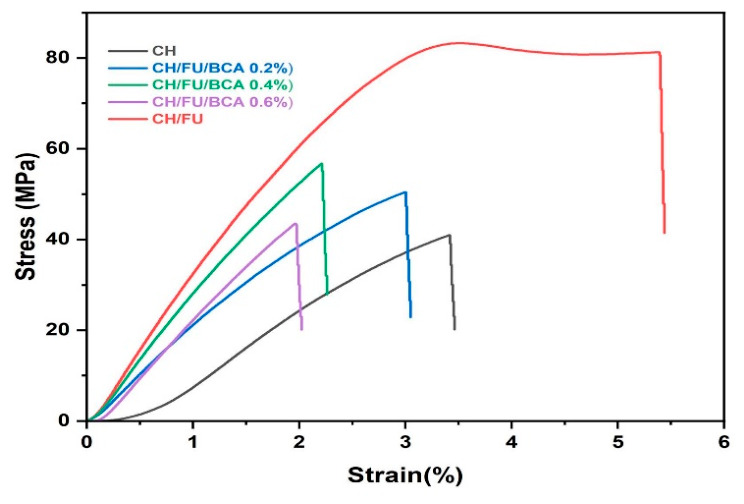
Stress–strain curves of CH, CH/FU, and CH/FU/BCA film samples.

The stress–strain curves presented in Figure 5 illustrate the mechanical behavior of the films under applied stress, reflecting the corresponding deformation (strain) response of the materials.

The tensile strength of pure chitosan film was used as a baseline for evaluating the effects of fucoidan and anthocyanin incorporation. Fucoidan, a sulfated polysaccharide, contributed to the enhancement of the mechanical properties of chitosan-based films. Its incorporation increased the ductility and stress resistance of the films, likely due to intermolecular interactions between the two polymers, resulting in the formation of a more robust network structure. The incorporation of blackcurrant anthocyanins (BCA) at varying concentrations (0.2%, 0.4%, and 0.6%) was investigated to evaluate their influence on the mechanical properties of the composite films. Anthocyanins are capable of interacting with the polymer matrix, thereby affecting the stress resistance of the films. The results indicated that BCA concentration exerted a concentration-dependent effect on mechanical performance. At 0.2% BCA, a slight variation in stress resistance was observed compared to the CH/FU film, suggesting a limited reinforcing or plasticizing effect [50]. Increasing the BCA content to 0.4% resulted in improved mechanical properties or a plateau in stress resistance, indicating an optimal balance between reinforcement and potential plasticization effects. In contrast, a further increase to 0.6% BCA led to a reduction in mechanical strength, which may be attributed to excessive incorporation disrupting the polymer network. The decline in mechanical performance at higher BCA concentrations may also be associated with the formation of aggregates or heterogeneous distribution within the film matrix, leading to stress concentration points that facilitate failure under applied load. Overall, the results demonstrate that the mechanical properties of chitosan–fucoidan films are significantly influenced by BCA incorporation, with 0.4% BCA providing the most favorable balance between reinforcement and structural integrity.

### 2.7. Zeta Potential

**Figure 6:**
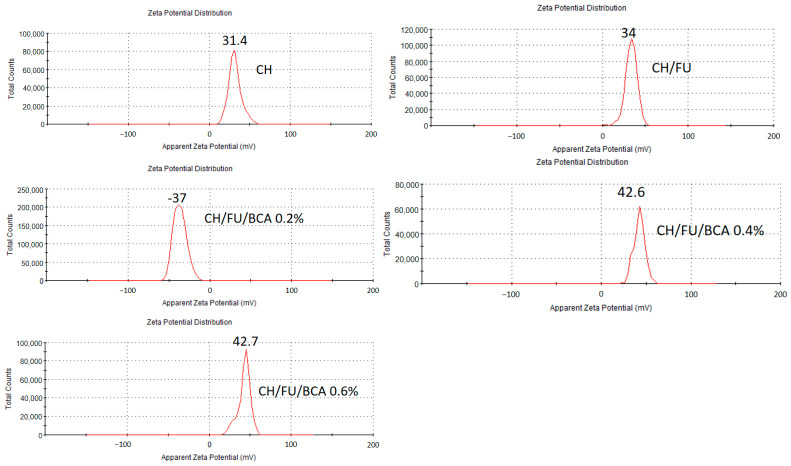
Zeta potential profiles of CH, CH/FU, and CH/FU/BCA film samples.

Figure 6 presents the zeta potential analysis, which provides insight into the stability and electrostatic interactions of chitosan (CH)-based films incorporated with fucoidan (FU) and blackcurrant anthocyanins (BCA). The results demonstrated that the addition of fucoidan and BCA increased the zeta potential from 31.4 mV for the pure chitosan film to 42.7 mV for the CH/FU/BCA film containing 0.6% BCA, indicating enhanced colloidal stability. Fucoidan, a sulfated polysaccharide, introduces negatively charged sulfate (–OSO_3_^−^) groups, while BCA, rich in phenolic compounds, contributes additional ionizable functional groups, thereby increasing the overall surface charge. The observed increase in zeta potential suggests a synergistic interaction among chitosan, fucoidan, and BCA, resulting in a higher charge density within the system. In general, systems with high absolute zeta potential values exhibit strong electrostatic repulsion between particles, which reduces aggregation and enhances stability. Conversely, low zeta potential values are associated with reduced electrostatic repulsion, increasing the likelihood of particle aggregation or flocculation. These results indicate that the incorporation of fucoidan and BCA improves the electrostatic stability of the composite films.

Anthocyanins contribute to system stability through hydrogen bonding interactions with chitosan and fucoidan. In addition, hydrophobic interactions between the aromatic rings of BCA and the nonpolar regions of the polysaccharide matrix further reinforce the film structure [51]. The relatively high zeta potential values (>±30 mV) confirm that the CH/FU/BCA films possess good colloidal stability, thereby reducing the likelihood of phase separation or flocculation [52]. Such stability is particularly advantageous for food packaging applications, where uniform dispersion of bioactive compounds and long-term structural integrity are critical.

### 2.8. Color Analysis

The color and visual appearance of edible films are critical factors influencing their functionality and consumer acceptance. As shown in Table 2, the color properties of the films were evaluated using L* (lightness), a* (red–green), and b* (yellow–blue) parameters, along with the whiteness index (WI) and total color difference (ΔE). Different lowercase letters indicate significant differences among the means within the same column (*p* < 0.05). The CIE Lab* color space encompasses all perceivable colors. In this system, L* stands for lightness, where 0 is black and 100 is white. Meanwhile, a* shows the shift between green (negative) and red/magenta (positive), and b* reflects the continuum from blue (negative) to yellow (positive) [53].

No notable differences could be identified between the pure chitosan (CH) films and chitosan–fucoidan (CH/FU) films in terms of total color difference (ΔE) and whiteness index (WI), indicating that the incorporation of fucoidan did not markedly affect the visual appearance of the films at the tested concentrations. In contrast, the incorporation of blackcurrant anthocyanins (BCA) resulted in an increase in WI and induced a color shift toward the red region, which can be attributed to the intrinsic pigmentation of anthocyanins.

### 2.9. Water Contact Angle; Water Vapor Permeability and Antimicrobial Activity

- Water Contact Angle

**Figure 7:**
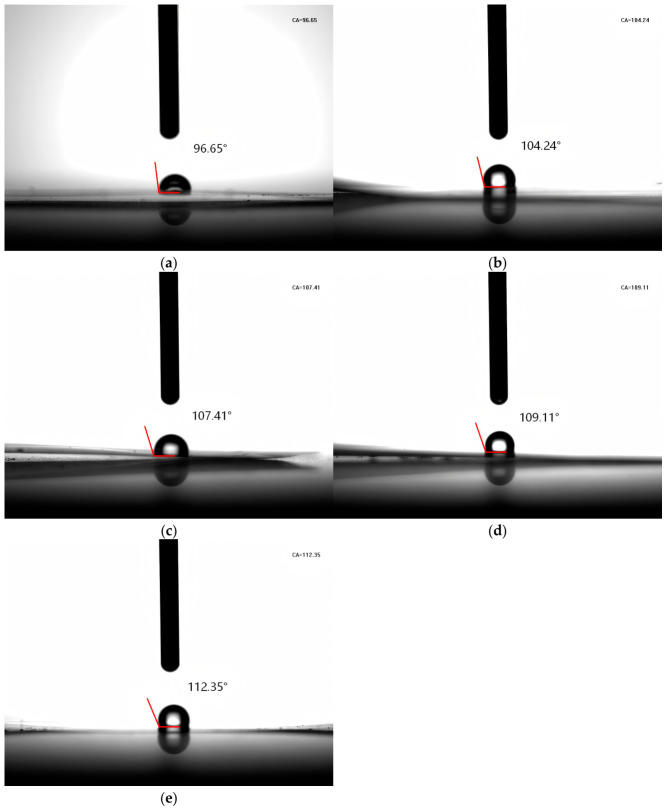
Water contact angle of (**a**) CH, (**b**) CH/FU, (**c**) CH/FU/BCA 0.2%, (**d**) CH/FU/BCA 0.4%, and (**e**) CH/FU/BCA 0.6%film samples.

The water contact angle (WCA) results are presented in Figure 7. In general, surfaces with a WCA lower than 65° are considered hydrophilic, whereas those with a WCA higher than 65° are classified as hydrophobic. As shown in Figure 7, the incorporation of BCA led to a noticeable increase in WCA, rising from 96.65° for the CH film to 107.41°, 109.11°, and 112.35° for CH/FU/BCA films containing 0.2%, 0.4%, and 0.6% BCA, respectively. This progressive increase in WCA indicates an enhancement in surface hydrophobicity, which can be attributed to the relatively hydrophobic nature of BCA. As observed in Figure 7c–e, the gradual increase in WCA with increasing BCA concentration further supports this trend.

- Water vapor permeability WVP

**Figure 8:**
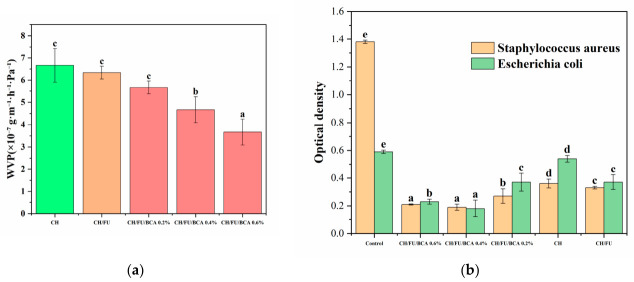
Water vapor permeability (**a**) and optical density (**b**) of CH, CH/FU, and CH/FU/BCA film samples. Different lowercase letters indicate significant differences (*p* < 0.05).

The water vapor permeability (WVP) values for the film samples are shown in Figure 8a. The WVP of the pure chitosan (CH) film was 6.66 ± 0.76, which decreased to 6.33 ± 0.28 in the CH/FU film. Such a slight WVP decline reflected the positive effect of FU incorporation on improving the water vapor barrier of chitosan-based films. This reduction can be explained by the diminished interaction between water molecules and the hydrophilic functional groups (–NH and –OH) of chitosan, resulting from electrostatic interactions and hydrogen bonding within the composite matrix [54]. Further incorporation of BCA led to a significant decrease in WVP, reaching 3.66 ± 0.57 for the CH/FU/BCA film containing 0.6% BCA. The WVP reduction became much more obvious with the introduction of BCA, implying a combined effect of FU and BCA on restricting water vapor penetration. This pronounced reduction may be associated with the formation of additional hydrogen bonds between BCA and the polymer matrix, which reduces the availability of hydrophilic sites and limits water vapor diffusion through the films. The constructed compact network structure further lengthened the diffusion route of water molecules, thereby further lowering the WVP of the composite films.

- Antimicrobial activity

Two representative bacterial strains—Gram-positive *Staphylococcus aureus* and Gram-negative *Escherichia coli*—were used to evaluate the antimicrobial activity of the coated films. Optical density (OD), which serves as an indicator of microbial growth (lower OD values correspond to stronger antimicrobial inhibition), is presented in Figure 8b. The control groups (without film) exhibited high OD values, indicating substantial bacterial growth. The results further compare the antimicrobial performance of chitosan (CH), chitosan–fucoidan (CH/FU), and CH/FU/BCA films against both bacterial strains.

The antimicrobial efficacy of the CH/FU/BCA films increased with rising BCA concentrations (0.6% > 0.4% > 0.2%), demonstrating enhanced inhibition against both bacterial strains. Specifically, *S. aureus* exhibited an OD value of 0.213 at 0.6% BCA, compared to 0.277 at 0.2%, while *E. coli* showed a reduction from 0.379 at 0.2% to 0.233 at 0.6%. Notably, the 0.4% BCA formulation exhibited slightly stronger inhibition against *S. aureus* than the 0.6% formulation suggesting a deviation from the general trend. This phenomenon is mainly attributed to the self-aggregation of excess anthocyanin molecules at 0.6% BCA, which weakens the dispersion and effective action of bioactive components. In comparison, 0.4% BCA achieves optimal dispersion and compatibility in the CH/FU matrix, enabling sufficient contact with bacteria and thereby presenting better antibacterial performance. Pure chitosan (CH) demonstrated comparatively weaker antimicrobial activity, with an OD value of 0.363 against *S. aureus*, whereas the CH/FU/BCA films exhibited significantly lower OD values, indicating enhanced antibacterial performance. The CH/FU film showed a similar level of inhibition to the low-dose (0.2%) BCA formulation, suggesting that BCA plays a key role in enhancing antimicrobial efficacy. The improved antimicrobial activity of the CH/FU/BCA system can be attributed to synergistic interactions among its components. BCA may facilitate the dispersion and interaction of CH and FU within the matrix, potentially improving their contact with bacterial cells and enhancing membrane disruption [55]. This effect is particularly pronounced against *S. aureus* (Gram-positive), which possesses a more accessible peptidoglycan layer, making it more susceptible to structural damage [56]. In contrast, *E. coli* (Gram-negative) exhibits greater resistance due to the presence of a lipopolysaccharide outer membrane that acts as a protective barrier. Overall, the concentration-dependent behavior suggests that 0.4% BCA provides an optimal balance between antimicrobial efficacy and structural stability, possibly by maximizing the availability of active components without inducing aggregation. These findings highlight the potential of CH/FU/BCA composite films as effective antimicrobial materials, particularly against Gram-positive bacteria.

### 2.10. Freshness and Preservation of Tuna

Fish is particularly prone to deterioration, driven by both endogenous enzymatic activity and microbial growth. During storage, the accumulation of degradation products leads to the formation of alkaline compounds, which increases the pH within the packaging system [57]. Therefore, pH, total volatile basic nitrogen (TVB-N), and thiobarbituric acid (TBA) values are commonly used as key indicators to evaluate fish freshness and quality, as presented in Table 3, Table 4 and Table 5.

**Figure 9:**
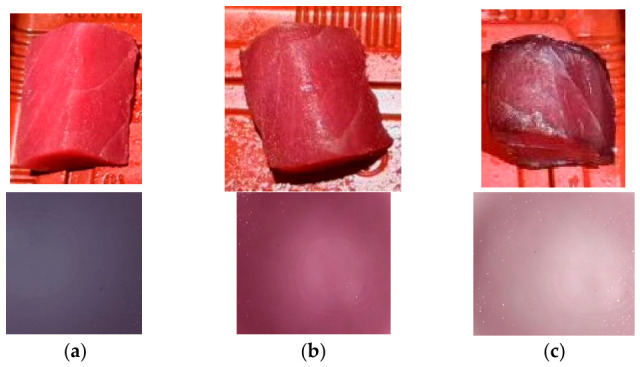
The Color of the CH/FU/BCA Corresponding to the Freshness of Tuna. (**a**) Fresh tuna; (**b**) Tuna at initial spoilage; and (**c**) Completely spoiled tuna.

Notably, the as-prepared composite films were employed to coat and wrap the surface of tuna, and exhibited distinct color changes that corresponded well to variations in tuna freshness. When the tuna remained in a fresh state, the composite film maintained a stable characteristic color, as shown in Figure 9a. As the tuna began to undergo initial spoilage, the film underwent an obvious color transition, as shown in Figure 9b. When the tuna was completely deteriorated and spoiled, the film further presented a remarkable color difference, as shown in Figure 9c.

The initial pH level of the tuna was around 6.7 on day 2. Across all groups, the pH showed a slight decrease: from day 2 to day 5 for the chitosan (CH) group, from day 2 to day 7 for the CH/FU group, and from day 2 to day 6 for the CH/FU/BCA 0.6% group, before rising during the later storage period. This early pH drop could be due to lactic acid in the fish flesh. Conversely, a pH increase might result from the buildup of alkaline substances (e.g., ammonia and trimethylamine) arising from microbial metabolism in the course of fish spoilage [58]. For the 0.2% and 0.4% CH/FU/BCA treatments, the pH continued to decline from day 2 to day 8, resulting in the lowest pH values across all groups—with the 0.2% CH/FU/BCA group reaching a minimum of 5.5. This indicates that CH/FU/BCA coatings significantly reduced alkaline compound formation by inhibiting microbial spoilage, particularly in comparison with pure chitosan. The incorporation of 0.2% and 0.4% BCA further enhanced this inhibitory effect.

Fish and shellfish are particularly prone to deterioration, and their quality is influenced by factors such as species, living environment, and feeding habits, together with the enzymatic and microbial activity within their tissues. Total volatile basic nitrogen (TVB-N), which consists of ammonia (NH_3_), dimethylamine (DMA), and trimethylamine (TMA), is a widely recognized indicator for assessing seafood quality and the freshness of marine fish. After death, the activity of spoilage bacteria increases, leading to the accumulation of TMA; thus, increases in TVB-N are typically associated with elevated TMA levels and total bacterial counts. TVB-N values are commonly used to classify fish freshness into four categories: very fresh (<10 mg N%), fresh (10–20 mg N%), acceptable for consumption (20–30 mg N%), and spoiled (>30 mg N%, unsafe for consumption). For tuna coated with CH/FU/BCA films, the TVB-N values on day 8 were 26.7, 25.6, and 25.9 mg N% for 0.2%, 0.4%, and 0.6% BCA, respectively, indicating that the samples remained within the acceptable range for consumption. A comparable trend was reported by Yu et al., who developed chitosan/corn starch films incorporating Vaccinium vitis-idaea anthocyanins. Their results demonstrated reduced TVB-N values, maintaining shrimp in a moderately fresh condition (17.5 mg/100 g) by day 6 [59].

Thiobarbituric acid (TBA) values are used to assess lipid oxidation, with higher values indicating more extensive oxidative deterioration. Over the 8-day storage period, the TBA values of the control group (pure chitosan, CH) and the CH/FU group increased more rapidly than those of the other treatments. In contrast, the CH/FU/BCA groups exhibited the slowest increase in TBA values. By day 8, the TBA values of the 0.2%, 0.4%, and 0.6% CH/FU/BCA groups remained comparatively low, at 0.58, 0.55, and 0.54 mg/kg, respectively. This reduction is likely attributable to the CH/FU/BCA film acting as an effective oxygen barrier, thereby slowing lipid oxidation in the fish samples.

## 3. Conclusions

The present study reports the development of an intelligent packaging film capable of responding to pH changes. The film was fabricated through self-assembly driven by hydrogen bonding and electrostatic interactions between chitosan and fucoidan, with BCA incorporated as an active component. The variation in microscopic morphology was closely correlated with the solubility, mechanical strength and water vapor permeability of the composite films. The intermolecular non-covalent interactions also facilitated the formation of a stable hydrogel network, laying a structural foundation for the subsequent casting and film-forming process. The inclusion of BCA imparted distinct pH-sensitive color transitions, ranging from red (pH 2–3) to purple (pH 7–9) and yellow (pH 10–12), enabling real-time monitoring of food freshness. Structural characterization by FT-IR and XRD confirmed the presence of hydrogen bonding interactions, which enhanced film stability without altering crystallinity. Morphological observations indicated that increasing BCA concentration (0.2–0.6%) resulted in smoother and more compact film structures. Such a dense and homogeneous microstructure effectively restricted water molecule infiltration, thereby reducing film solubility and water vapor permeability, and simultaneously supporting the improvement of mechanical strength. Thermal analysis demonstrated improved resistance to degradation. The films also exhibited enhanced hydrophobicity, with a maximum water contact angle of 112.35°, along with reduced water solubility (7.02%) and improved barrier performance, as evidenced by water vapor permeability being reduced by 45%. Mechanical properties were optimized at 0.4% BCA, providing a balanced combination of flexibility and strength. The optimal microstructure at appropriate BCA loading contributed to balanced flexibility and mechanical robustness, further verifying the inherent relationship between structural morphology and macroscopic functional properties. The films exhibited antimicrobial activity against *Staphylococcus aureus* and *Escherichia coli*, with the strongest inhibition at 0.4% BCA. In tuna preservation tests, the films effectively delayed spoilage, maintaining relatively low TVB-N values (25.6–26.7 mg N%) and TBA values (0.54–0.58 mg/kg) over an 8-day storage period. Overall, the CH/FU/BCA films demonstrated potential as an environmentally friendly packaging material, integrating pH-responsive indicators with improved barrier, mechanical, and antimicrobial properties for effective seafood freshness monitoring. However, several limitations remain. The long-term stability of pH-responsive color changes has not yet been evaluated, and food safety assessments are incomplete. Further studies should include migration analysis and cytotoxicity testing to assess potential leaching and biocompatibility for food contact applications. In addition, future work should address scale-up production, evaluate performance across different food systems, and investigate consumer acceptance. These efforts are necessary to support the practical application of this technology in reducing food waste and improving food safety.

## 4. Materials and Methods

### 4.1. Materials

Blackcurrant anthocyanin (BCA) was obtained from New Zealand Extracts Ltd., Marlborough, New Zealand. Fucoidan (fucose content ≥ 95%, CAS No. 9072–19–9, molecular weight ~15 kDa) and chitosan (CAS No. 9012–76–4, molecular weight ~150 kDa, viscosity 100–200 mPa·s, degree of deacetylation 95%) were purchased from Shanghai McLean Reagent Co., Shanghai, China. *Escherichia coli* (AC12194) and *Staphylococcus aureus* (ATCC 25923) were obtained from ACMEC Biochemical Shanghai, China. All other chemical reagents employed throughout this study met analytical grade specifications.

### 4.2. Characterization of Blackcurrant Anthocyanin (BCA)

The Folin–Ciocalteu assay was employed to measure the total phenolic content (TPC) present in BCA [60]. BCA was combined with Folin–Ciocalteu reagent and 10% sodium carbonate solution, and the mixture was kept in the dark for 60 min to allow full color development. Absorbance was then determined at 765 nm, and the final content was calculated and expressed as milligrams of gallic acid per gram of extract. All measurements were performed in triplicate, and TPC was expressed as mg gallic acid (GA) per g of extract. The antioxidant activity of BCA was evaluated via ABTS and DPPH radical scavenging assays. Briefly, 2 mL of BCA solution (1 mg/mL) was mixed with 2 mL of DPPH solution (0.15 mM) and incubated for 30 min at 517 nm, or with 2 mL of ABTS^+^ solution (0.15 mM) for 15 min at 734 nm, after which absorbance was measured.

The anthocyanin content of BCA was quantified using the pH differential method [61]. BCA was dissolved in buffer solutions at pH 1.0 and 4.5, and the absorbance of each solution (1 mg/mL) was recorded at 520 and 700 nm. The differential absorbance (A) was calculated as:

A = (A_520nm_ − A_700nm_)_at pH=1_ − (A_520nm_ − A_700nm_)_at pH=4.5_ (1)

the total anthocyanin content (mg/g) was determined according to the following equation:

Total anthocyanin content (mg/g) = A × MW × DF × V × 1000/m × ε(2)

where MW is the molecular weight of anthocyanin (449.2 g/mol), DF is the dilution factor, V is the volume of extract (mL), m is the weight of BCA (g), and ε is the molar absorptivity of cyanidin-3-glucoside (26,900 L·mol^−1^·cm^−1^).

To investigate pH-dependent color stability, BCA solutions (1 mL, 1 mg/mL) were added to 4 mL of buffer solutions spanning pH 2–11 and incubated for 3 min. The resulting color changes were documented photographically. Additionally, UV–Vis absorption spectra of BCA in the different pH buffers were measured across a wavelength range of 450–700 nm [62].

### 4.3. Film Preparation

Chitosan-based films were prepared following the methods of Wang, F. et al. and Afshar, S.V. & Boldrin, A. [41,63], with minor modifications. Chitosan (1.6 g) was dissolved in 80 mL of 1% (*v*/*v*) acetic acid and stirred at 80 °C for 60 min. An 80 mL aliquot of this chitosan solution was cast onto a 20 cm × 20 cm plexiglass plate to form the control film (CH). Fucoidan (1.0 g) was dissolved in 100 mL of distilled water and stirred at 80 °C for 10 min. Subsequently, 15 mL of the fucoidan solution was mixed with 80 mL of the chitosan solution and stirred at 80 °C for 5 min. After cooling to approximately 50 °C, blackcurrant anthocyanin (BCA) was incorporated at varying concentrations (0.2, 0.4, and 0.6 wt% relative to chitosan) and stirred for 60 min at ambient temperature without heating.

**Figure 10:**
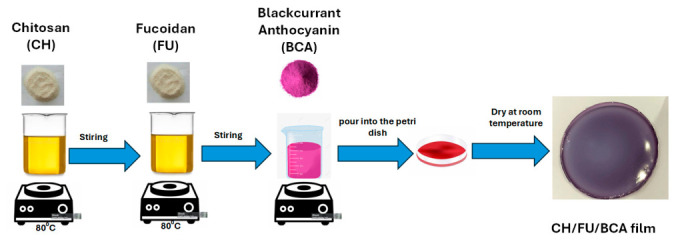
Schematic overview of the preparation protocol for CH/FU/BCA films.

The resulting film-forming solution was degassed using ultrasonication, cast onto plexiglass plates (20 cm × 20 cm), and dried at room temperature for 3 days. All films were subsequently stored in vacuum-sealed bags within a desiccator until further analysis. The pure chitosan film was designated as CH, the chitosan–fucoidan composite as CH/FU, and the chitosan–fucoidan films containing BCA at 0.2, 0.4, and 0.6 wt% were labeled as CH/FU/BCA 0.2%, CH/FU/BCA 0.4%, and CH/FU/BCA 0.6%, respectively. The preparation procedure of CH/FU/BCA composite films is shown in Figure 10.

### 4.4. Fourier Transform Infrared (FTIR) Spectroscopy

Blackcurrant anthocyanin (BCA) was mixed with potassium bromide (KBr) at a ratio of 1:100 in a mortar and subsequently dried under an infrared lamp for 10 min. Film samples were cut into 1 cm × 1 cm pieces and dried similarly for 30 min. FTIR spectra of the BCA and film samples were recorded using a Thermo Fisher Scientific spectrometer (Waltham, MA, USA) at a resolution of 4 cm^−1^ over the wavenumber range of 4000–500 cm^−1^ [64].

### 4.5. X-Ray Diffraction (XRD)

BCA and film samples were dried under an infrared lamp for 10 min prior to analysis. The X-ray diffraction profiles of the samples were acquired using a D8 Advance diffractometer (Bruker, Karlsruhe, Germany) at a scanning rate of 5°/min over a 2θ range of 10–40° [65].

### 4.6. Morphology Analysis

Film samples were lyophilized in the dark and subsequently sputter-coated with a thin layer of gold. The surface morphology was examined using a scanning electron microscope (SEM; Hitachi S-3400 N, Tokyo, Japan) at an accelerating voltage of 5.0 kV [66].

### 4.7. Thermal Characteristic Analysis

The thermal properties of the films were evaluated using a thermogravimetric analyzer (TGA; Pyris Diamond TG/DTA, PerkinElmer, Waltham, MA, USA). Samples were subjected to heating from 50 °C to 800 °C at a constant rate of 10 °C per minute under a nitrogen atmosphere maintained at a flow rate of 100 mL/min [67].

### 4.8. Film Thickness, Moisture Content (MC), and Water Solubility (WS)

Film thickness was measured using a digital micrometer (G-2.4 N, PEACOCK Ozaki, Tokyo, Japan). Moisture content (MC) was determined by drying the films at 105 °C for 24 h, and calculated as the percentage reduction in weight relative to the initial weight [68].

Water solubility (WS) was assessed following the method of Mohsen Pouralkhas et al. [69]. To determine solubility, 2.5 × 2.5 cm film samples were oven-dried at 105 °C for 24 h to establish a constant starting weight. They were then placed in 50 mL of distilled water for 24 h. Following immersion, the liquid was passed through Whatman No. 1 filter paper. The solid residue left on the filter was collected and re-dried at 105 °C for 24 h before being weighed. The solubility percentage was calculated based on the amount of mass lost compared to the initial weight. All measurements were conducted in triplicate.

Moisture content (%) = (Initial weight − Final weight)/Initial weight × 100

Water solubility (%) = (Initial weight − Final weight)/Initial weight × 100

### 4.9. Zeta Potential Measurements

Zeta potential measurements of CH/FU/BCA were performed at physiological pH and 25 °C using a Zetasizer Nano ZS (Malvern Instruments Ltd., Malvern, Worcestershire, UK). Electrophoretic mobility was analyzed using the Smoluchowski equation. All reported values are the mean of ten independent measurements [56].

### 4.10. Mechanical Properties

Mechanical properties were evaluated in accordance with the ASTM D882-18 standard [70]. Rectangular specimens (175 mm × 25 mm) were prepared from the film sheets and tested using a Universal Testing Machine (Model 3367, Instron Corporation, Norwood, MA, USA) equipped with a 1 kN load cell. Five specimens per treatment were analyzed in each trial. The initial grip separation was set at 125 mm, with a crosshead speed of 50 mm/min. Prior to testing, all specimens were conditioned for 48 h at 25 ± 2 °C and 50 ± 2% relative humidity, which were maintained throughout the measurements. Evaluated tensile properties included ultimate tensile strength (UTS, MPa), elongation at break (EB, %), and Young’s modulus (YM, MPa) [71].

### 4.11. Water Contact Angle (WCA)

To evaluate surface hydrophobicity, the water contact angle (WCA) of the films was measured using an optical contact angle meter (DataPhysics Instruments, Stuttgart, Germany) at room temperature. A 5 μL droplet of distilled water was gently placed on the film surface. Measurements were taken at multiple locations on each film to evaluate uniformity and barrier properties.

### 4.12. Water Vapor Permeability (WVP)

The water vapor permeability (WVP) of the film was assessed using a slightly modified version of the previous testing method by [72]. Film samples were tightly sealed over custom-designed aluminum cups (outer diameter: 8.0 cm; inner diameter: 6.8 cm), yielding an exposed film area of 36.3 cm^2^. Each cup, 5.5 cm in depth, was filled with 6 g of anhydrous CaCl_2_ and placed inside a desiccator maintained at 95% relative humidity at room temperature. The weight of each cup was recorded at 2 h intervals throughout the test.

(3) WVP=W×x/t×A×ΔP

WVP was calculated using the following parameters: W represents the weight gain during the measurement period (g), x denotes the average film thickness (m), t is the exposure time (h), A is the permeable film area (m^2^), and ΔP corresponds to the water vapor pressure difference (2340 Pa at 20 °C).

### 4.13. Color Analysis

Chitosan-based composite films were cut into 10 cm × 10 cm pieces and placed over blackcurrant anthocyanin for photographic documentation. Film transmittance was measured across the 200–800 nm wavelength range. Additionally, smaller film samples (2 cm × 2 cm) were immersed in citrate-phosphate buffer solutions with pH values ranging from 2 to 11 for 1 h, and the resulting color changes were recorded photographically. The total color difference (ΔE) and the whiteness index (WI) were estimated according to the equations described by [73].

(4) $$WI=100-\sqrt{{\left(100-L^{*}\right)}^{2}+a^{*2}+b^{*2}}$$

(5) $$\Delta E=\sqrt[{2}]{{\left({L_{0}}^{*}-L^{*}\right)}^{2}+{\left(a^{*}-a\right)}^{2}+{\left(b^{*}-b\right)}^{2}}$$

In this colorimetric system, L* signifies lightness, a* represents the chromatic axis from red to green, and b* denotes the axis from yellow to blue, allowing quantitative evaluation of color variations in response to pH changes.

### 4.14. Antimicrobial Activity

The antibacterial efficacy of the composite films was evaluated using the enzyme-labeled kinetic method [74]. The prepared composite films were extracted with sterile solution, and the extracts were applied to bacterial cultures for antibacterial assessment. *Staphylococcus aureus* and *Escherichia coli* cultures were grown to the logarithmic phase and subsequently transferred into sterilized LB medium. All film samples and bacterial inocula were stored at 4 °C before use to ensure stability. The cultures were incubated for 24 h at 31 °C under dark conditions with agitation at 150 rpm. Subsequently, 20 µL of the film extract was added to each well, and 100 µL samples were collected and transferred to 96-well microplates. The absorbance at 600 nm was measured continuously using a microplate reader. Five treatment groups (CH, CH/FU, and CH/FU/BCA at 0.2%, 0.4%, and 0.6%) and a control group were set. All measurements were performed in triplicate. The experimental design included a control group and five treatment groups, each with three replicates.

### 4.15. Application in Tuna Preservation and Freshness Assessment

- Thiobarbituric acid (TBA) assay

Five grams of tuna tissue was accurately weighed and mixed with 25 mL of 7.5% trichloroacetic acid containing 0.1% ethylenediaminetetraacetic acid. The mixture was homogenized at 10,000 rpm for 15 s and subsequently centrifuged at 10,000 rpm and 4 °C for 10 min. A 5 mL portion of the supernatant was mixed with 5 mL of 0.02 mol/L TBA solution and heated in a boiling water bath for 30 min. Following cooling, the mixture was centrifuged at 5000 rpm for 5 min. The absorbance of the supernatant obtained was recorded at 532 nm using an enzyme-linked immunosorbent assay (ELISA) reader. TBA values were expressed as malondialdehyde (mg MDA/kg), with a standard curve prepared using tetraethoxypropane at concentrations of 0–40 µmol/L [75].

- Total volatile basic nitrogen (TVB-N) assay

Ten grams of tuna tissue were accurately weighed and homogenized with 100 mL of deionized water. After filtration, 5 mL of 10 g/L magnesia solution was added to the filtrate. Steam distillation was carried out for 3 min using an automatic Kjeldahl analyzer(Drawell Scientific Instrument Co., Ltd., Shanghai, China), and the distillate was absorbed by 10 mL of 20 g/L boric acid solution. The resulting solution was titrated with 0.01 mol/L hydrochloric acid, and the TVB-N content was expressed as mg per 100 g of tuna sample [76].

- pH measurement

An appropriate amount of tuna tissue was accurately weighed and homogenized with distilled water at a 1:10 (g:mL) ratio. The homogenate was allowed to stand for 10 min and subsequently filtered. The pH of the filtrate obtained from homogenizing 5 g of tuna tissue in 50 mL of distilled water was measured at ambient temperature using a calibrated pH meter. All experiment were conducted in triplicate, with daily measurements of all five treatment groups recorded over 8 days of storage.

### 4.16. Statistical Analysis

The properties of chitosan films containing fucoidan and varying concentrations of anthocyanins were evaluated using a standard chitosan film as the control. Each experiment was conducted in triplicate to ensure accuracy. One-way analysis of variance (ANOVA) was employed for data analysis, subsequently complemented by Duncan’s multiple range test, using a significance criterion of *p* < 0.05. Graphical representations were generated using OriginLab 2024b software. Results are presented as mean ± standard deviation (SD).

## Figures and Tables

**Table 1:** Moisture content (MC), thickness, and water solubility (WS) of CH, CH/FU, and CH/FU/BCA film samples.

Treatment	Thickness	Moisture Content	Water Solubility
CH	0.036 ± 0.017 ^a^	16.7 ± 0.056 ^b^	16.22 ± 0.020 ^c^
CH/FU	0.029 ± 0.001 ^a^	11.4 ± 0.080 ^a^	12.34 ± 0.002 ^b^
CH/FU/BCA 0.2%	0.038 ± 0.001 ^a^	10.4 ± 0.042 ^a^	11.38 ± 0.002 ^b^
CH/FU/BCA 0.4%	0.034 ± 0.001 ^a^	9.5 ± 0.040 ^a^	11.29 ± 0.002 ^b^
CH/FU/BCA 0.6%	0.034 ± 0.001 ^a^	8.4 ± 0.037 ^a^	7.02 ± 0.001 ^a^

**Table 2:** Color parameters (L*, a*, b*, whiteness index (WI), and total color difference (ΔE)) of CH, CH/FU, and CH/FU/BCA film samples.

Treatments	L*	a*	b*	WI	ΔE
CH	64.41 ± 0.39 ^b^	−0.15 ± 0.04 ^c^	1.71 ± 0.31 ^c^	35.62 ± 0.31 ^a^	control
CH/FU	62.78 ± 5.30 ^b^	−0.05 ± 0.17 ^c^	2.10 ± 0.47 ^c^	37.27 ± 5.36 ^a^	6.09 ± 0.41 ^a^
CH/FU/BCA0.2%	61.24 ± 3.01 ^b^	−0.79 ± 0.18 ^a^	−1.40 ± 0.25 ^b^	38.79 ± 3.02 ^a^	6.22 ± 0.24 ^a^
CH/FU/BCA0.4%	52.37 ± 2.28 ^a^	−0.68 ± 0.14 ^a^	−2.53 ± 0.62 ^a^	45.90 ± 3.49 ^b^	7.32 ± 0.19 ^b^
CH/FU/BCA0.6%	46.37 ± 2.89 ^a^	−0.56 ± 0.12 ^a^	−3.08 ± 0.27 ^a^	53.72 ± 2.90 ^b^	8.74 ± 0.35 ^b^

**Table 3:** pH values of CH, CH/FU, and CH/FU/BCA. Different lowercase letters indicate statistically significant differences (*p* = 0.05).

Treatments	D2	D3	D4	D5	D6	D7	D8
CH	6.9 ± 0.06 ^b^	6.7 ± 0.12 ^b^	6.1 ± 0.10 ^a^	5.5 ± 0.10 ^a^	6.3 ± 0.34 ^c^	6.6 ± 0.10 ^c^	7 ± 0.15 ^e^
CH/FU	6.8 ± 0.10 ^ab^	6.7 ± 0.15 ^b^	6.4 ± 0.23 ^bc^	6.1 ± 0.15 ^c^	5.9 ± 0.25 ^b^	5.6 ± 0.12 ^a^	5.9 ± 0.10 ^c^
CH/FU/BCA 0.2%	6.9 ± 0.21 ^b^	6.7 ± 0.25 ^b^	6.5 ± 0.06 ^c^	6.3 ± 0.10 ^d^	5.9 ± 0.12 ^b^	5.7 ± 0.20 ^a^	5.5 ± 0.21 ^a^
CH/FU/BCA 0.4%	6.8 ± 0.11 ^ab^	6.6 ± 0.12 ^b^	6.3 ± 0.21 ^b^	6.1 ± 0.11 ^c^	6 ± 0.25 ^b^	5.9 ± 0.23 ^b^	5.7 ± 0.10 ^b^
CH/FU/BCA 0.6%	6.7 ± 0.15 ^a^	6.3 ± 0.31 ^a^	6.1 ± 0.10 ^a^	5.9 ± 0.12 ^b^	5.6 ± 0.10 ^a^	5.9 ± 0.15 ^b^	6.1 ± 0.23 ^d^

**Table 4:** TVB-N (mg N/100 g) values of CH, CH/FU, and CH/FU/BCA. Different lowercase letters indicate statistically significant differences (*p* = 0.05).

Treatments	D2	D3	D4	D5	D6	D7	D8
CH	13.9 ± 0.95 ^a^	17.6 ± 1.22 ^b^	25.3 ± 1.70 ^c^	28.1 ± 1.67 ^c^	35.7 ± 1.13 ^c^	38.2 ± 1.74 ^d^	46.9 ± 1.39 ^c^
CH/FU	13.7 ± 1.28 ^a^	16.9 ± 1.62 ^b^	21.1 ± 1.29 ^b^	24.6 ± 1.88 ^b^	29.2 ± 1.23 ^b^	31.8 ± 1.12 ^c^	34.7 ± 3.19 ^b^
CH/FU/BCA 0.2%	13.8 ± 0.25 ^a^	14.6 ± 0.96 ^a^	15.6 ± 1.56 ^a^	18.9 ± 1.76 ^a^	23.7 ± 1.48 ^a^	28.8 ± 1.67 ^b^	26.7 ± 1.34 ^a^
CH/FU/BCA 0.4%	13.8 ± 1.14 ^a^	14.9 ± 1.48 ^a^	15.6 ± 1.32 ^a^	18.3 ± 1.39 ^a^	22 ± 1.78 ^a^	24.4 ± 2.17 ^a^	25.6 ± 1.15 ^a^
CH/FU/BCA 0.6%	13.7 ± 0.84 ^a^	14.7 ± 1.73 ^a^	15.8 ± 1.49 ^a^	18.5 ± 1.09 ^a^	22.4 ± 2.26 ^a^	24.6 ± 1.37 ^a^	25.9 ± 0.57 ^a^

**Table 5:** TBA (mg MDA/kg) values of CH, CH/FU, and CH/FU/BCA. Different lowercase letters indicate statistically significant differences (*p* = 0.05).

Treatments	D2	D3	D4	D5	D6	D7	D8
CH	0.28 ± 0.01 ^b^	0.34 ± 0.03 ^c^	0.46 ± 0.06 ^c^	0.55 ± 0.05 ^d^	0.69 ± 0.09 ^d^	0.71 ± 0.12 ^d^	0.74 ± 0.14 ^d^
CH/FU	0.19 ± 0.03 ^a^	0.29 ± 0.04 ^b^	0.38 ± 0.01 ^b^	0.44 ± 0.08 ^c^	0.51 ± 0.05 ^c^	0.57 ± 0.02 ^c^	0.62 ± 0.06 ^c^
CH/FU/BCA 0.2%	0.18 ± 0.02 ^a^	0.26 ± 0.08 ^a^	0.35 ± 0.07 ^a^	0.39 ± 0.05 ^a^	0.43 ± 0.09 ^a^	0.49 ± 0.06 ^a^	0.58 ± 0.04 ^b^
CH/FU/BCA 0.4%	0.18 ± 0.03 ^a^	0.27 ± 0.04 ^a^	0.34 ± 0.04 ^a^	0.38 ± 0.02 ^a^	0.44 ± 0.06 ^a^	0.51 ± 0.05 ^ab^	0.55 ± 0.03 ^a^
CH/FU/BCA 0.6%	0.19 ± 0.05 ^a^	0.27 ± 0.09 ^a^	0.38 ± 0.06 ^b^	0.42 ± 0.03 ^b^	0.45 ± 0.05 ^b^	0.48 ± 0.02 ^b^	0.54 ± 0.08 ^a^

## Data Availability

The original contributions presented in this study are included in the article. Further inquiries can be directed to the corresponding authors.
